# Inactivation of mediator complex protein 22 in podocytes results in intracellular vacuole formation, podocyte loss and premature death

**DOI:** 10.1038/s41598-020-76870-0

**Published:** 2020-11-18

**Authors:** Patricia Q. Rodriguez, David Unnersjö-Jess, Sonia S. Zambrano, Jing Guo, Katja Möller-Hackbarth, Hans Blom, Timo Jahnukainen, Lwaki Ebarasi, Jaakko Patrakka

**Affiliations:** 1 Integrated Cardio Metabolic Center, Division of Pathology, Department of Laboratory Medicine, Karolinska Institutet, Karolinska University Hospital Huddinge, Stockholm, Sweden; 2 Science for Life Laboratory, Department of Applied Physics, Royal Institute of Technology, Solna, Sweden; 3 Department of Medical Biochemistry and Biophysics, Karolinska Institutet, Stockholm, Sweden; 4 Cardiovascular and Metabolic Disorders Program, Duke-NUS Medical School, Singapore, Singapore; 5 Department of Pediatric Nephrology and Transplantation, New Children’s Hospital, Helsinki University Hospital, University of Helsinki, Helsinki, Finland

**Keywords:** Nephrons, Mechanisms of disease, Molecular biology, Nephrology

## Abstract

Podocytes are critical for the maintenance of kidney ultrafiltration barrier and play a key role in the progression of glomerular diseases. Although mediator complex proteins have been shown to be important for many physiological and pathological processes, their role in kidney tissue has not been studied. In this study, we identified a mediator complex protein 22 (Med22) as a renal podocyte cell-enriched molecule. Podocyte-specific Med22 knockout mouse showed that Med22 was not needed for normal podocyte maturation. However, it was critical for the maintenance of podocyte health as the mice developed progressive glomerular disease and died due to renal failure. Detailed morphological analyses showed that Med22-deficiency in podocytes resulted in intracellular vacuole formation followed by podocyte loss. Moreover, Med22-deficiency in younger mice promoted the progression of glomerular disease, suggesting Med22-mediated processes may have a role in the development of glomerulopathies. This study shows for the first time that mediator complex has a critical role in kidney physiology.

## Introduction

Renal podocyte cells are terminally differentiated epithelial cells of the glomerulus in where they form the final ultrafiltration barrier of the kidney^1^. Podocytes are morphologically divided into three compartments: cell bodies, major and foot processes^2^. Large cell bodies are located in the urinary space from which they extend large cellular protrusion, major processes, towards glomerular capillaries. Close to the capillary wall, the cellular extensions divide to finely interdigitating foot processes that wrap around capillaries. The foot processes are interconnected by a specialized cell–cell junction called the slit diaphragm. In most glomerular diseases, the slit diaphragm and/or foot processes of podocytes are injured^1^. Morphologically, this is observed as foot process effacement^2^. This is considered to be a key event in the development of albuminuria^2^, which, on the other hand, is a cardinal sign of kidney disease. Foot process effacement is a reversible process mediated by actin-rich cytoskeleton of foot processes, and it is a common down-stream effect of various pathological stimuli^2^. However, in progressive renal diseases, the effacement may lead to loss of podocytes^3^.

Overwhelming evidence pinpoints podocytes as central players in the pathogenesis of glomerulopathies and chronic kidney disease (CKD)^1^. One of the key reasons for this is that podocytes are terminally differentiated and cannot proliferate^1^. Studies in human diseases have shown that podocyte loss correlates with the progression rate of glomerular disease^4,5^. Studies in mouse have validated the key pathogenic role of podocytes as overexpression or inactivation of genes specifically in podocytes can either aggravate or slow the development of CKD^6,7^. Obviously, understanding mechanisms that govern podocyte homeostasis is of crucial importance.

The Mediator complex (MED) is a multi-protein assembly that acts as a transcriptional co-activator in all eukaryotes^8^. The complex is composed of at least 26 subunits in mammals, and its function is to communicate signals from DNA-bound transcription factors to the RNA polymerase II enzyme. Different transcription factors bind to different mediator subunits and many transcription factors can simultaneously bind to MED^8^. An important feature of MED is that its subunit composition can vary^8^. Consequently, the lack of individual MED subunits is associated with silencing of specific transcription pathways. Studies in knockout animals have unravelled roles of MED subunits in cellular differentiation and studies in human genetics have linked them to a number of human diseases. However, to date, no studies on MED in kidney tissue have been performed.

In this study, we explored MED subunits in renal podocyte cells. We show that MED subunit 22 (Med22) is highly enriched in podocytes and although it is not needed for the normal development of podocytes, it is essential for the maintenance of glomerular homeostasis as mice lacking Med22 in podocytes develop progressive renal disease and die prematurely.

## Results

### Human Protein Atlas suggests Med22 as a podocyte-associated MED subunit

As the role of MED in kidney tissue is unknown, we analysed Human Protein Atlas (www.proteinatlas.org) to identify podocyte-associated MED subunits. We found immunohistochemical data in kidney tissue for 34 MED subunits (Suppl. Figure 1). Med21 and Med22 showed strong glomerular staining with only low signal in tubuli. As Med22 mRNA seemed to give more glomerulus-specific staining (Suppl. Figure 1), we focused on Med22 in further studies.

**Figure 1:**
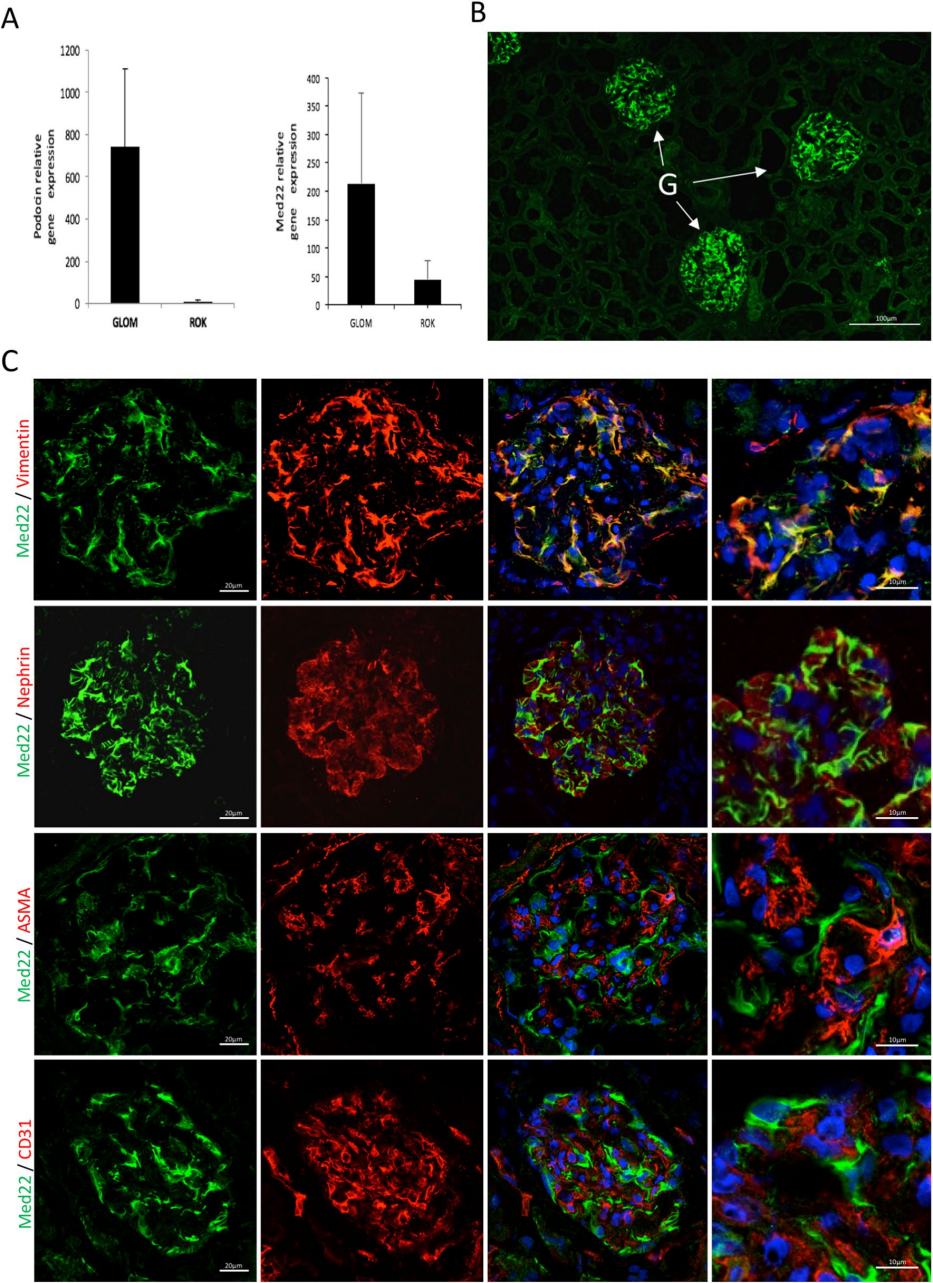
Med22 is expressed highly by podocytes. (**A**) qPCR analysis of human kidney tissue shows that Med22 is expressed more strongly by glomerular tissue (glom) than rest of kidney fraction (rok). Podocin, a podocyte-specific gene, was used to control purity of glomerular fractions. (**B**) Immunofluorescence staining for Med22 in adult human kidney shows strong signal in glomeruli (G) and only sparse reactivity outside glomeruli. (**C**) Double labelling experiments with vimentin (red) shows partial co-localization (yellow) in podocyte major processes. No significant overlapping reactivity is observed with foot process marker nephrin (red), mesangial marker alpha-smooth muscle actin (ASMA, red) or endothelial marker CD31 (red).

### Med22 is enriched in podocytes and localizes to major processes

In the kidney, the expression of Med22 was enriched in the glomerulus in comparison to the kidney fraction devoid of glomeruli as shown by qPCR human kidney tissue (Fig. 1A). The expression of podocyte-specific gene podocin^9^ was analysed to control the purity of glomerulus fractions (Fig. 1A). In immunofluorescence of human kidney tissue a strong reactivity for Med22 was detected in glomeruli with clearly weaker signal in rest of kidney tissue (Fig. 1B). In double stainings, Med22 co-localized partially with podocyte major process marker vimentin (Fig. 1C). No significant overlap was detected with podocyte foot process marker nephrin, mesangial cell expressed protein alpha-smooth muscle actin and endothelial marker CD31 (Fig. 1C). Moreover, Med22 did not co-localize significantly with a podocyte nucleus marker Wt1 (Suppl. Figure 2A). Taken together, Med22 staining was in the glomerulus detected in podocytes in which it located to major processes. The specificity of anti-Med22 antibody was validated by immunostaining and Western blotting of cultured podocytes transfected with full-length human Med22 cDNA (Suppl. Figure 2B,C).

**Figure 2:**
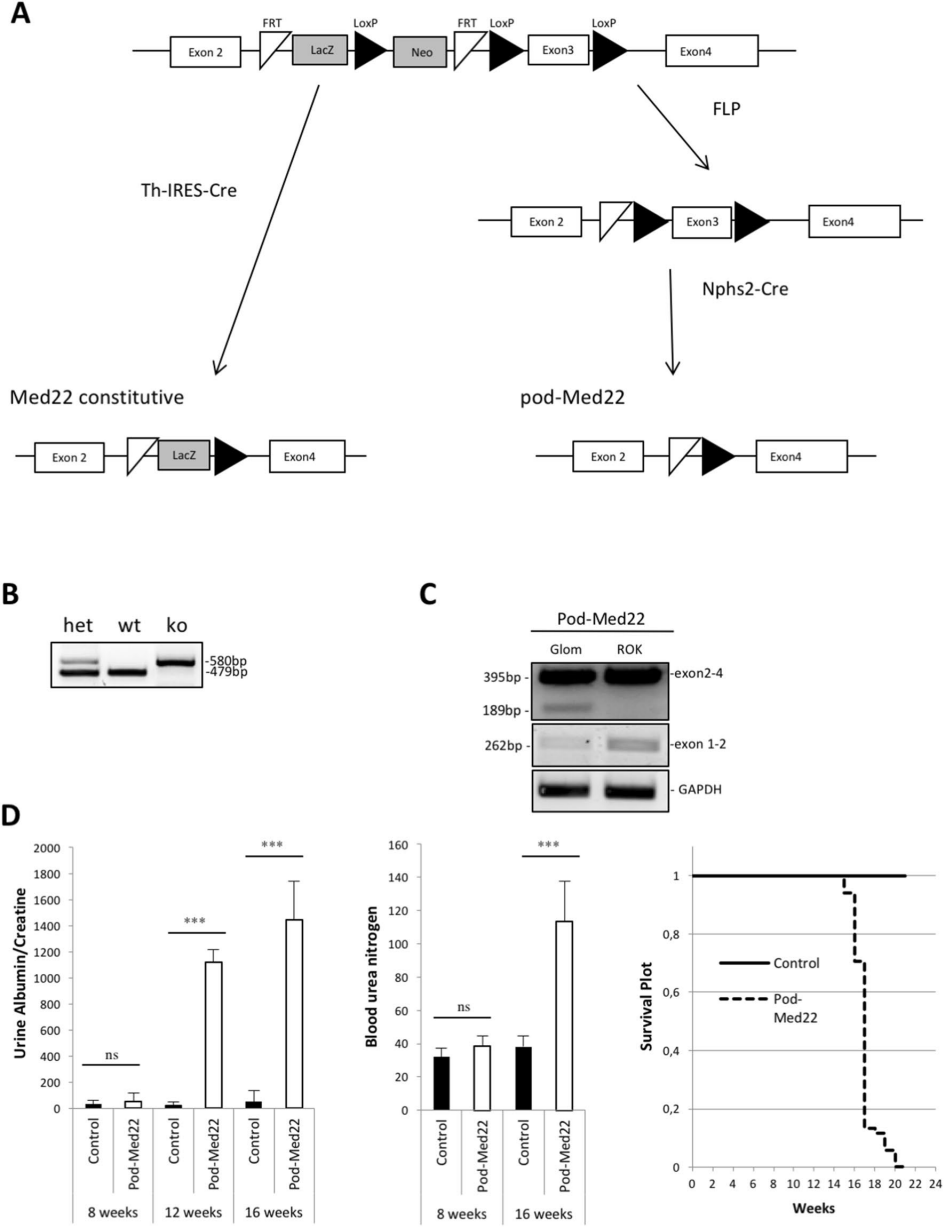
Generation and characterization of podocyte-specific Med22 mouse line. (**A**) A mouse line carrying a cassette with lacZ and neomycin-resistance genes, as well as FRT and LoxP sites targeting the exon 3 of the Med22 gene, was crossed with ThIRES-Cre line to generate a conventional knockout allele, and with a FLP-deleter line to generate a conditional knockout allele. Floxed mouse line was crossed with a NPHS2-cre line to generate podocyte-specific knockout line. (**B**) Genotyping of conditional allele generated a 580 bp product, whereas wild type produced a 479 bp band. (**C**) In control mice, Med22 mRNA spanning exon 2–4 and exon 1–2 were amplified in glomerular, lung and spleen tissue. In pod-Med22 mice, the same products were amplified but also an additional shorter variant in glomerular tissue was detected with primers targeting exons 2 and 4. This corresponded in size to the skipping of exon 3. (**D**) No significant albuminuria is detected in pod-Med22 mice at 8 weeks of age as detected by measuring urine albumin/creatinine ratios. At 12 weeks, pod-Med22 mice show massive albuminuria that further progresses by 16 weeks of age. Blood urea nitrogen levels were unchanged in pod-Med22 mice at 8 weeks of age but were significantly elevated at 16 weeks of age. Pod-Med22 mice start to die prematurely at 15 weeks of age and no mice survive past 20 weeks. *ns* not significant, **p < 0.01, ***p < 0.001.

### Generation of constitutive and conditional Med22 knockout mouse lines

To analyse the role of Med22 in the glomerulus, we first generated a conventional knockout (KO) mouse line. A transgenic mouse line with a cassette containing lacZ and neomycin-resistance genes, along with FRT and LoxP sites targeting the exon 3 of the Med22 gene, was obtained from European Mouse Mutant Cell Repository. This line was crossed with ThIRES-cre mouse line that is active in oocytes^10^ to generate a germ line deletion and thus a constitutive Med22 allele lacking exon 3 (Fig. 2A). Breeding of animals heterozygous for constitutive allele did not generate any homozygous mice (166 pups analysed), indicating embryonic lethality. To see whether haploinsufficiency of Med22 resulted in glomerular diseases, we followed heterozygous mice for up to 15 months. No abnormalities were detected in histological or blood/urine analysis (Suppl. Figure 3A,B). Therefore, we decided to generate a conditional Med22 allele.

**Figure 3:**
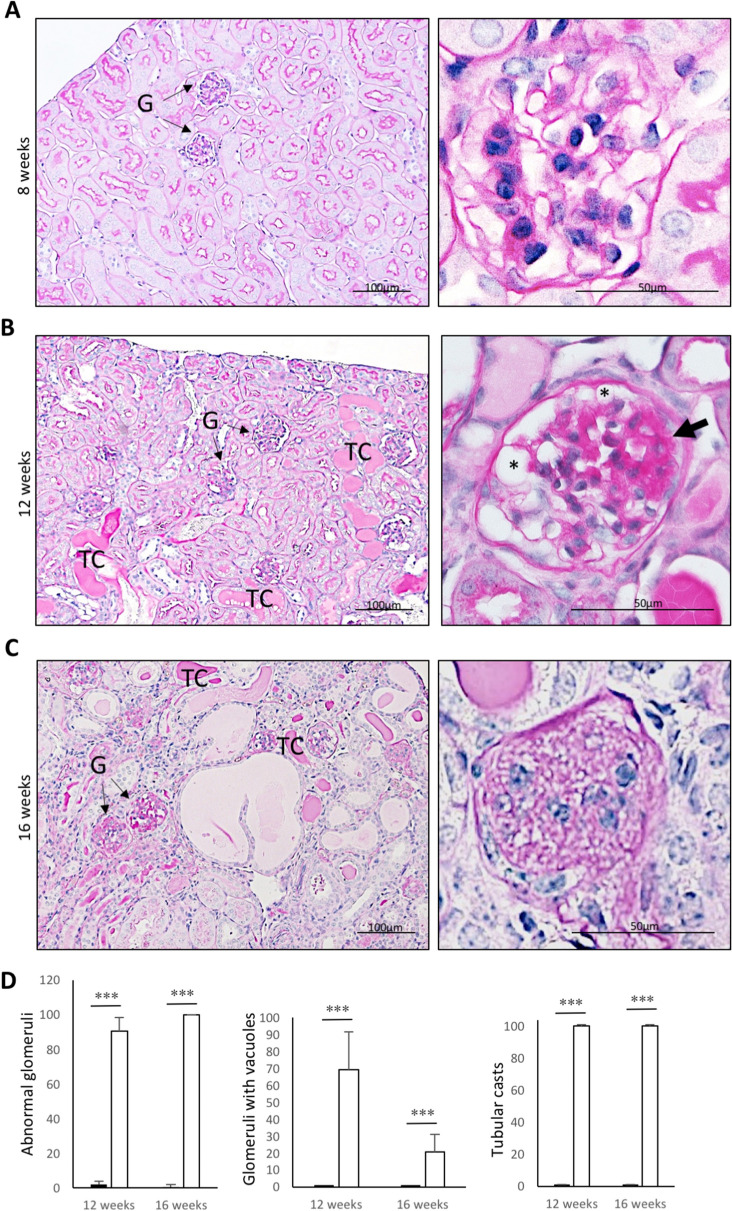
Histological analysis of pod-Med22 mice show formation of vesicular structures in podocytes followed by development of glomerulosclerosis. (**A**) No histological abnormalities are detected in 8-week old mice as shown by PAS staining. (**B**) At 12 weeks, numerous glomerular and tubulointerstitial abnormalities are detected. In glomeruli, focal segmental sclerosis (arrow) is detected and large vesicular structures (*) are observed. Tubuli show hyaline casts and dilation, and interstitial fibrosis is observed. (**C**) At 16 weeks of age, most glomeruli are completely sclerotic. Tubuli show more pronounced dilation and hyaline casts. (**D**) Semi-quantification of histological changes in pod-Med22 mice versus littermate control mice. There is a significant increase in a proportion of abnormal glomeruli, the presence of glomerular vacuoles and tubular casts at both 12 and 16 weeks of age. *G* glomerulus; *TC* tubular casts. ***p < 0.001.

To inactivate Med22 specifically in podocytes, we crossed the original transgenic line with a FLP-deleter line followed by crossing with a Nphs2-cre line (Fig. 2A)^11^. Genotyping for this allele amplified 580 bp product, whereas the wild type product was 479 bp (Fig. 2B). To validate the successful deletion of exon 3 we analyzed Med22 mRNA in isolated glomeruli. In controls, we amplified a single cDNA fragment spanning from exon 2 to exon 4 (Fig. 2C). In mice with the conditional allele, we detected a shorter variant corresponding in size to skipping of exon 3 in isolated glomeruli, whereas this variant was not amplified from rest of kidney tissue (Fig. 2C). We confirmed the loss of exon 3 in glomerular tissue by Sanger sequencing (data not shown). As part of the Med22 mRNA seemed intact, we termed this mouse line as a podocyte-specific Med22 mutant line (pod-Med22) that can represent either a null or a hypomorphic allele.

### Podocyte-specific defect in Med22 results in vacuole formation, podocyte loss and progressive renal disease

Pod-Med22 mice were born in a normal Mendelian ratio and developed normally (data not shown). No albuminuria was detected by 8 weeks of age (Fig. 2D). However, the mice exhibited massive albuminuria by 12 weeks of age that further increased by 16 weeks of age. In line with this, animals showed normal blood urea nitrogen levels at 8 weeks of age but the levels were significantly increased at 16 weeks of age (Fig. 2D). All pod-Med22 animals (a total of 14) died by 20 weeks of age due to renal failure, whereas littermate control animals (a total of 64), including both wild type and heterozygote mice, showed 100% survival (Fig. 2D).

In light microscopy, 4- and 8-week old pod-Med22 kidneys showed no abnormalities (Fig. 3A, data not shown). At 12 weeks of age, pod-Med22 mice exhibited large “empty” vacuole-like structures in podocytes that were often difficult to discern from capillary lumens in light microscopy (Fig. 3B). These structures were negative for lipids as detected by oil red staining (data not shown). Focal segmental glomerulosclerosis, which develops secondary to podocyte loss, was also observed (Fig. 3B). In addition, dilated tubuli with hyaline casts, a secondary sign of albuminuria, were detected (Fig. 3B). At 16 weeks of age, more advanced changes were detected with almost global glomerulosclerosis and tubular atrophy (Fig. 3C). Semi-quantification of key histological features validated the abundant histological changes of pod-Med22 mice (Fig. 3D).

**Figure 4:**
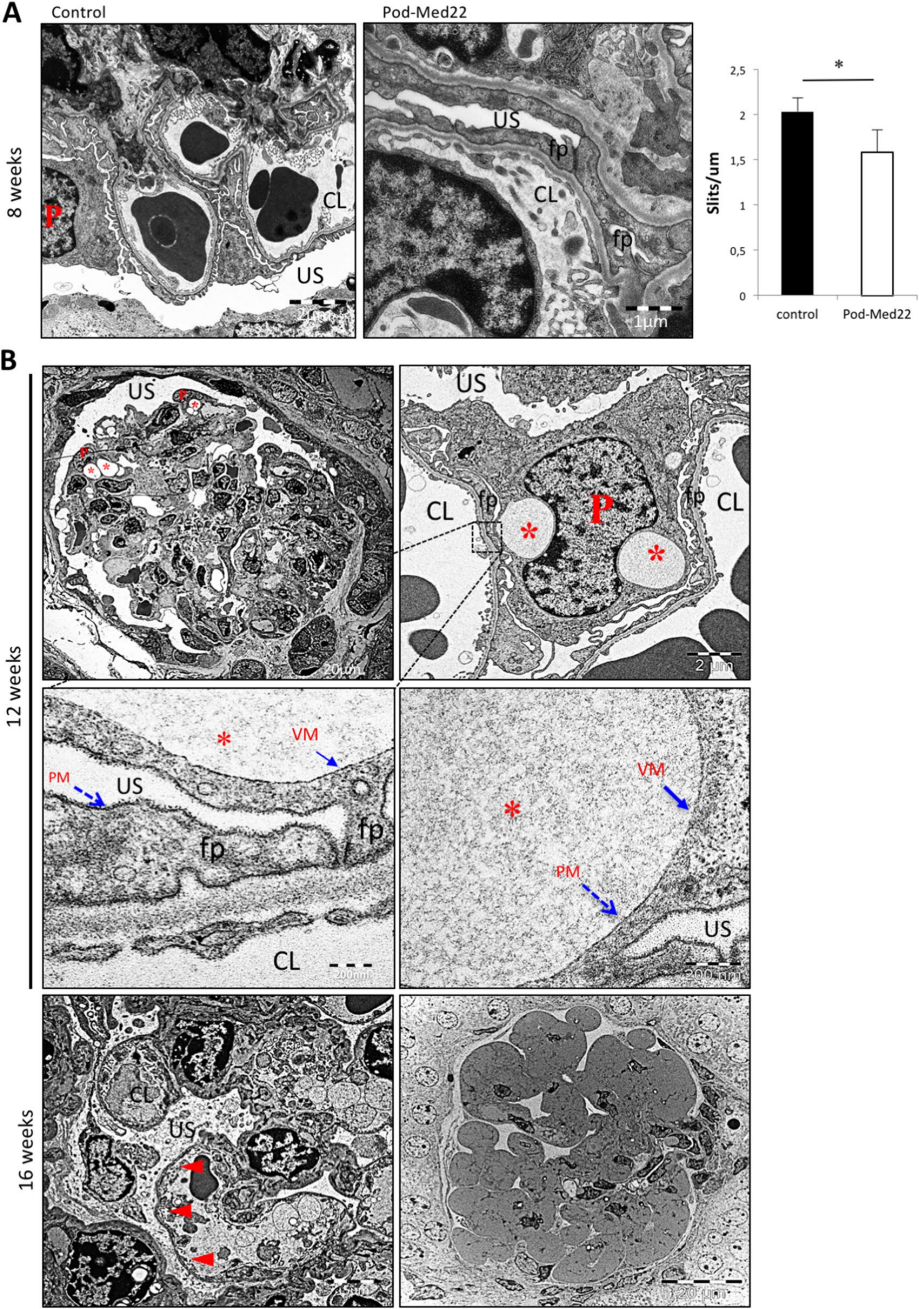
Electron microscopy of pod-Med22 kidneys shows progressive vesicular lesions in podocytes. (**A**) At 8 weeks, pod-Med22 mice show podocyte foot process effacement as validated by calculating number of slits per GBM length. (**B**) At 12 weeks, vesicular structures (*) are regularly detected in podocyte (P) cytoplasm. Occasionally, the vesicles are obstructing podocyte nuclei. Vesicular membranes (VM) lack electron-dense surface that is present on plasma membrane (PM) of podocytes. (**C**) At 16 weeks of age, glomeruli show areas of GBM that are not covered by podocytes (arrowheads) indicating podocyte loss. Moreover, completely sclerotic glomeruli are detected. *US *urinary space; *CL* capillary lumen; *fp *foot process. *p < 0.05.

In electron microscopy, glomeruli appeared normal in 4-week old mice but at 8 weeks of age pod-Med22 mice showed a significant foot process effacement (Fig. 4A). Rarely, small vacuoles in podocytes were observed in 8-week old pod-Med22 mice (data not shown). At 12 weeks of age, round vacuoles were regularly observed inside podocytes (Fig. 4B). These vacuoles showed a single simple membrane, which lacked the electron-dense glycocalyx that was detectable on podocyte plasma membrane (Fig. 4B). Vacuoles showed occasionally heterogeneous electron density, which was in contrast to urinary space that demonstrated mostly clear homogenous signal. Occasionally, podocytes showed nuclear changes suggesting an apoptotic process (Fig. 4B). At 16 weeks of age, sclerotic regions in glomeruli and occasionally areas of the glomerular basement membrane (GBM) that were not covered by podocytes were detected (Fig. 4B), suggesting the loss of podocytes.

As vacuole-like structures reminded of a sub-podocyte space^3,12^, we performed a high-resolution microscopy and detailed electron microscopic analysis to see whether these structures had connections to Bowman’s space. In pod-Med22 podocytes carrying a tomato-reporter, we detected spherical structures that were surrounded by cytoplasmic (Suppl. Media 1 and 2). A detailed visual evaluation of these structures showed that only 12 out of 77 vacuoles (16%) showed a suspected connection to Bowman’s space (Suppl. Media 1 and 2). In electron microscopy, the analysis of vacuole-like structures in 40 pod-Med22 glomeruli (aged 12–16 weeks) showed no connections to Bowman’s space (data not shown). Taken together, morphological analysis showed that pod-Med22 mice develop intracellular vacuoles in podocytes followed by loss of podocytes.

### Immunofluorescence analysis of pod-Med22 podocytes

**Figure 5:**
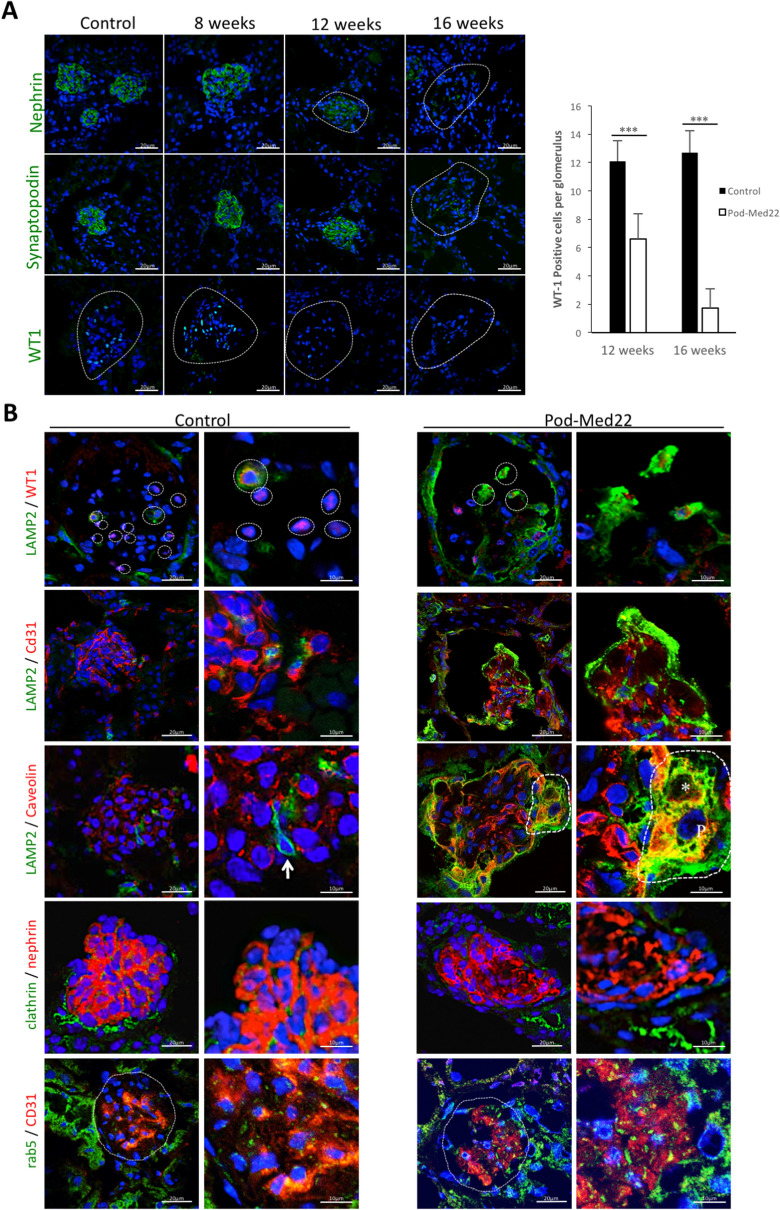
Immunofluorescence analysis of podocytes in pod-Med22 mice. (**A**) Expression of podocyte proteins nephrin, synaptopodin and WT1 progressively decreases in pod-Med22 glomeruli. At 8-weeks, staining for podocyte markers is similar to control animals. In 12-week old mice, the signal is clearly decreased and at 16-weeks, nephrin and WT1 staining is rarely detected and only occasionally reactivity for synaptopodin is observed. Quantification of WT1 positive nuclei per glomerulus validates the loss of podocytes. (**B**) In pod-Med22 mice, strong up-regulation of LAMP2 is detected in WT1-positive cells. Double labelling with CD31 shows that LAMP2 is located on the urinary side of CD31 staining and no overlapping reactivity is detected. Immunoreactivity for caveolin is clearly increased in LAMP2 positive cells in pod-Med22 glomeruli. Clathrin and Rab5 do not show any differential expression in pod-Med22 glomeruli. ***p < 0.001.

To analyse podocytes in more detail, we stained for podocyte markers nephrin, synaptopodin and WT1. All markers showed progressive loss of signal starting at 8 weeks and by 16 weeks only few glomeruli showed positivity (Fig. 5A). The quantification of WT1 nuclei showed a clear reduction at both 12 and 16 weeks. The results are in line with our morphological data that suggest dedifferentiation followed by loss of podocytes.

To investigate the molecular nature of podocyte vacuoles, we performed immunofluorescence staining for vesicular markers. Lysosomal marker LAMP2 was upregulated in 12-week old glomeruli and localized mainly to podocytes as shown by double-labelling with WT1 and CD31 (Fig. 5B). Of note, LAMP2 was not up-regulated in 8-week old pod-Med22 glomeruli (data not shown). Endocytosis marker caveolin was also upregulated in podocytes at 12 weeks as caveolin co-localized with LAMP2 (Fig. 5B). Neither endosomal marker clathrin nor early endocytosis marker Rab5 were changed in comparison to controls (Fig. 5B). Moreover, no difference in autophagy markers beclin, Atg16L, Atg5 and LC3A/B was detected by immunofluorescence or Western blot (data not shown).

### RNAseq of KO glomeruli unravels downregulation of podocyte genes

To get molecular insights into the process in pod-Med22 glomeruli, we performed RNA sequencing (RNAseq) on glomeruli isolated from 8-week old mice. The sequencing revealed 126 significantly differentially expressed genes of which 78 were up- and 48 downregulated (Suppl. Table 1). There was a down-regulation of podocyte genes, which was in line with our immunofluorescence data. Besides downregulation of Rab3b, no vesicle transport genes were differentially expressed.

### Pod-Med22 mice are susceptible to kidney damage

**Figure 6:**
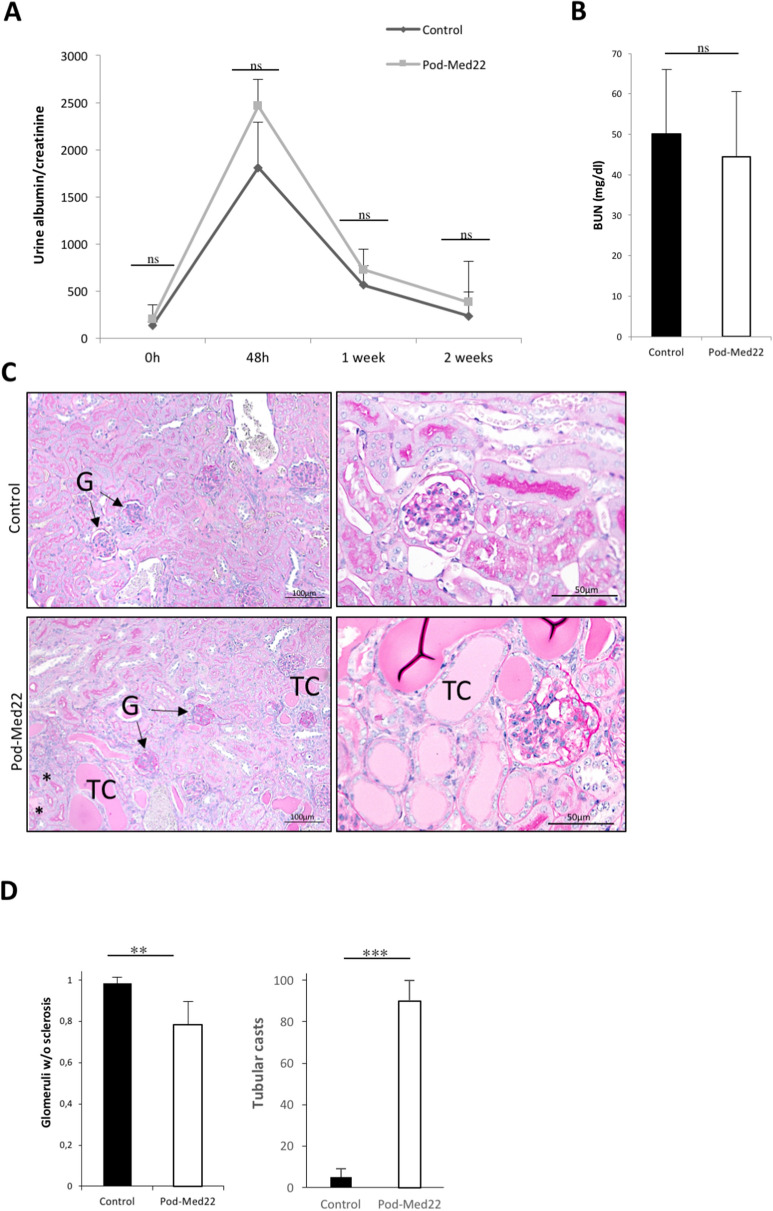
Pod-Med22 podocytes are sensitive to stress and respond by vesicle formation. (**A**) After induction of glomerular injury by nephrotoxic serum, both pod-Med22 and control mice develop massive albuminuria and no significant difference is detected between the groups. (**B**,**C**) In histological analysis 14 days after the induction of glomerulonephritis, significantly more sclerotic lesions were detected in pod-Med22 mice. Similarly, significantly more tubular casts are detected in pod-Med22 mice. *G *glomerulus; *TC* tubular cast. *ns *not significant, **p < 0.01, ***p < 0.001.

As pod-Med22 animals did not show any renal phenotype during the first 8 weeks of life, we wanted to analyse whether a Med22-defect in podocytes modulated disease progression during this period. We induced glomerulonephritis at 4 weeks of age using a nephrotoxic serum that binds to the GBM. Both pod-Med22 and litter-mate controls developed similar albuminuria within 48 h after the induction (Fig. 6A). No significant difference in BUN levels were detected (Fig. 6B). However, in histological examination we detected a significant difference as pod-Med22 mice developed more glomerular damage as indicated by scoring of sclerotic changes (Fig. 6C,D). Additionally, more tubular casts were detected in pod-Med22 kidneys (Fig. 6C,D).

## Discussion

We identified mediator complex protein Med22 as a podocyte-enriched molecule and show that although Med22 is not needed for normal podocyte development, it plays a critical role in the maintenance of glomerular filtration barrier as mice with a mutant Med22 in podocytes develop a progressive renal disease. Our study shows for the first time that the mediator complex has a critical role in kidney biology.

So far, only few studies have focused on Med22. It was originally termed as Surf5, due to its location in Surfeit locus in mouse genome^13^, and later identified to be a component of the mediator complex, and possibly to directly interact with Med30^14^. Interestingly, studies in cell culture suggested that Med22 protein is localized in the cytoplasm^15^. Our immunofluorescence studies support this notion as we detected the Med22 protein mainly in the cytoplasm of podocytes. This was somewhat surprising taking into account the role of MED in transcription. It may be that Med22 shuttles between the cytoplasm and the nucleus, similar to MED subunit 28, which has been suggested to have dual functions in these compartments^16,17^. Due to lack of Med22 expression in podocyte cell culture models, we did not investigate this further.

Med22-deficiency in mouse podocytes resulted in renal failure and premature death by 20 weeks of age. Starting around 8 weeks-of-age mice developed intracellular vacuoles, which was followed by podocyte death. The exact mechanism how these vesicles lead to podocyte death is unclear, but our temporal morphological analysis showed that the vesicles grew dramatically in size and finally occupied almost the whole cytoplasm displacing nuclei and other intracellular structures. This can result in general cellular dysfunction and cell death.

Mediator is a multi-protein complex that is essential for gene transcription via RNA polymerase II^8,18^. So far, all KO mouse models generated for mediator subunits have been embryonic lethal suggesting that mediator action is critical for embryonic development^8,18^. Our study supports this notion as embryos homozygous for constitutive Med22 allele die in utero. Embryonic lethality has hampered in vivo studies on mediator complex. To overcome this, we inactivated Med22 specifically in podocytes and saw that although Med22 was highly expressed by these cells, it was not essential for the development or initial maintenance of podocyte structure and function. There are several potential explanations for this. It is possible that Med22-dependent biological processes become active later in life. In fact, although the glomerulus seems to be structurally mature 2 weeks after birth in mouse, we have observed significant expressional differences in isolated glomeruli at later stages of life (unpublished data). On the other hand, it is possible that Med22 allele generated is not a null but a hypomorphic allele and this function could be sufficient for normal podocyte development. However, we speculate that the “late” phenotype may be a result of the accumulation of damage. Minor defects in transcriptional machinery may exist already at birth but these can fall under our detection threshold. This idea is supported by our data in 4-week-old pod-Med22 mice that were prone to develop podocyte damage and developed vacuole-like structures under pathological stimuli.

We made an effort through RNAseq and immunostaining to pinpoint molecular mechanisms that drive the disease development in pod-Med22 mice. However, we failed to identify a clear pathway that would be responsible for the phenotype. It may be that Med22 mediates transcription of multiple pathways that are difficult to define through molecular profiling. Thus, it is difficult to say whether Med22-mediated pathways contribute to the pathogenesis of human glomerular diseases. Vacuole-like structures in podocytes, reminding of the phenotype in our pod-Med22 mice, have been reported in common human glomerulopathies^19,20^, and therefore, it is possible that Med22 has a role even in human glomerulopathies. Obviously, more studies are needed to dissect the role of Med22 in podocytes, as well as to understand how mediator complex contributes to kidney biology.

## Materials and methods

### RT-PCR

Glomeruli were isolated from human and mouse kidneys as published previously^21,22^. RT-PCR experiments were performed using standard methods. Primer sequences for Med22 were: Human 5′-GTT GGG GAT CTC AGC AGT GG-3′, 5′-CAG CCC ATG GCA CAG ACA TA-3′. For the amplification of mouse exons, we used: Med22Ex1-2 CAG CCC TCA GTA CTC GGT TC (left), Med22Ex1-2 GCT CGA ACG TGC ATC TCA TA (right), Med22 Ex2-4 AGG TGT CTC GGG CTA CTC AG (left), Med22Ex2-4 TAA GGG ACC AGC ACC AGT CT (right).

### Immunofluorescence

Normal human adult kidney samples were collected from kidneys nephrectomised due to renal cancer (Karolinska University Hospital, Stockholm, Sweden). Nephrin and podocin antibodies have been described previously^9,23^. Other antibodies were: Med22—Atlas Antibodies and Sigma; Vimentin, alpha SMA, WT-1—Sigma (human); CD31—Abcam; Clathrin, Caveolin, Lc3II, Atg16L, Atg5, Beclin, Rab5, Rab7, Rab11 (Cell Signalling); Synaptopodin, Rab3b and LAMP2 (Santa Cruz), mouse nephrin (Acris), mouse WT1 (Millipore).

The samples were snap-frozen, and the cryosections (10 μm) were post-fixed with cold acetone (− 20 °C) followed by blocking in 5% normal goat serum. The primary antibodies were incubated overnight at 4 °C, followed by a 1-h incubation with the secondary antibody. For double-labelling experiments, the incubations were performed sequentially.

### Generation of Med22 knockout mouse lines

We used a line generated by European Mouse Mutant Cell Repositor**y** in which the cassette containing lacZ, neomycin-resistance gene, and FRT and LoxP sites were targeted to exon 3 of Med22 gene. The mice were in a mixed C57bl/6 129 Sv background. We crossed these mice with Th-IRES deleter^10^, as well as with a FLP-deleter line (B6.129S4-Gt(ROSA)26Sortm1(FLP1)Dym/RainJ) to generate a floxed mouse line for Med22 gene (Med22-fl). Med22-fl was crossed Tg-Nphs2-cre^11^ to inactivate Med22 specifically in podocytes (pod-Med22).

### Histological analyses

Histological analyses were done using PAS (Periodic Acid-Schiff) staining. For 16-week-old mice, 8 controls and 8 knockout animals were analysed. For 12-week-old, 17 controls and 7 knockout animals were analysed. For 8-weeks-old, 7 controls and 6 knockout animals were analysed. For 1-year-old Med22 global heterozygous 4 controls and 7 heterozygous mice were analysed at 15 months. Glomerular damage was evaluated by semi-quantitative scoring of histological changes. Glomeruli were scored as having normal histology or abnormal histology (which was usually associated with the presence of sclerosis/mesangial matrix expansion/crescents). The presence of detectable vacuole-like structures in each glomeruli was evaluated and scored separately. We evaluated 30 random glomeruli in each mouse. For the analysis of tubular changes, we chose 10 random high power filed (× 40) images from renal cortex and observed the presence of tubular casts.

Electron microscopic analysis were with standard transmission microscopy using samples fixed with 2.5% glutaraldehyde. Especially, we focused on podocytes and vesicular structures in podocytes. A total of 40 vesicular structures in 12 and 16-week old pod-Med22 mice were analysed and categorized as having a connection to Bowman’s space or being isolated vesicle without detectable connection to extracellular space.

### Tissue expansion and analysis of expanded kidney samples

Kidney samples were expanded as described previously^24^. Samples were then stained for tdTomato using a goat anti-tdTomato antibody (Sigma AB8181-200) and a donkey anti-goat Alexa-488 secondary antibody. Samples were then imaged on a Zeiss LSM780 inverted confocal microscope using a 40X NA1.3 water immersion objective.

Every large vesicle in podocytes (exceeding 1 µm in depth) was categorized into three groups; open, closed or non-conclusive. Only vesicles that were completely surrounded by tdTomato signal were marked as closed. The vesicles that could not be completely excluded to have an opening to extracellular space were marked as non-conclusive. The vesicles with an apparent opening to extracellular space were marked as open.

### Urine analysis

Albuminuria was analysed by running 2 µl of urine on SDS-PAGE gel stained with PAGE-Blue stain (Invitrogen) or/and by calculating urinary albumin/creatinine ratios (Exocell Albuwell kit, Bioassay kit). Following animals were analysed: For 16-week-old mice 6 littermates and 5 pod-Med22 animals; For 12-week-old mice 14 littermates and 7 pod-Med22 animals; For 8-week-old mice 7 littermate and 6 pod-Med22 animals; For 4-week-old mice 5 littermates and 5 pod-Med22 animals.

### Blood analysis

Blood-urea-nitrogen (BUN) values were determined using ELISA kit from Abcam. Following mice were analysed: 5 pod-Med22 and littermate control animals at 16-weeks of age, 6 pod-Med22 and 9 littermate control animals at 8 weeks of age, as well as 5 pod-Med22 and 5 littermate control animals treated with nephrotoxic serum (see below).

### Bulk RNA sequencing

RNA sequencing was performed for glomeruli isolated from 3 pod-Med22 and 3 littermate control mice at 8-weeks of age. Total RNA was isolated using a combination of Trizol and Chloroform extraction and Qiagen RNAeasy clean-up columns. Total RNA quality and concentration were measured by Agilent BioAnalyzer 2100. All samples passed quality criteria RIN > 8.0, 28S/18S > 1.0. RNA samples were then prepared and sequenced by commercial service in BGI tech.

100 bp pair-end reads (pure reads without adapters) were received from BGI tech. More than 22 million reads passed quality control (quality score > 28) for each sample. Qualified reads were mapped to mouse reference genome Ensembl GRCm38 and transcriptome gene guild version 89 with alignment tool STAR 2.5.2b^25^. Uniquely mapped reads were then quantified as counts by FeatureCount v1.5.1. Differentially expressed genes were tested by R package DESeq2^26^. Only genes with significance FDR < 0.05 (False Discovery Rate) are reported.

### Anti-GBM glomerulonephritis model

The induction of glomerulonephritis using anti-glomerular serum (Probetex, cat: PTX-001S) was performed as recently described^27^. A total of 10 4-week old animals (5 pod-Med22 and 5 controls) were used.

Glomerular damage was evaluated by semi-quantitative scoring of histological changes. Glomeruli were scored as having normal histology or abnormal histology (which was usually associated with the presence of sclerosis/mesangial matrix expansion/crescents). We evaluated 30 random glomeruli in each mouse (5 control and 5 pod-Med22 mice). For the analysis of tubular changes, we chose 10 random high power filed (× 40) images from renal cortex and observed the presence of tubular casts.

### Statistical methods

We used Prism statistics program: Student T test parametric (even groups) and non-parametric (non-even groups). Minimum for significance was *p* < 0.05, in the figures *p* values are indicated as follows: *p < 0.05, **p < 0.01, ***p < 0.001.

### Ethical considerations

The use of human material for studies was approved by the local Ethics Review Authority (“Etikprövningsmyndighet”, www.epn.se) in Stockholm, Sweden, archive number 2017-58-31/4. Informed consent was obtained from all subjects. All methods used in human material were carried out in accordance with relevant guidelines and regulations defined in the ethical permit (see above).

For mouse work, all experimental protocols were approved by The Linköping Ethical Committee for Research Animals (“Linköpings djurförsöksetiska nämnd”), Linköping, Sweden (archive number DNR 41-15). All methods used in mouse experiments were carried out in accordance with relevant guidelines and regulations defined in the ethical permit (see above).

## Supplementary information

Supplementary Figures.

Supplementary Table 1.

Supplementary Movie 1.

Supplementary Movie 2.

Supplementary Legends.
