# Supplementary material for: Inactivation of mediator complex protein 22 in podocytes results in intracellular vacuole formation, podocyte loss and premature death
Source: Sci Rep. 2020 Nov 18;10:20037. doi: 10.1038/s41598-020-76870-0 (PMC7676236; doi:10.1038/s41598-020-76870-0)

Inactivation of mediator complex protein 22 in podocytes results in intracellular vacuole formation, podocyte loss and premature death

Patricia Q. Rodriguez, PhD[1], David Unnersjö-Jess PhD[2], Sonia S. Zambrano, PhD [1], Jing Guo J[3,4], Katja Möller-Hackbarth, PhD[1], Hans G. Blom, PhD[2], Timo Jahnukainen, MD[5], Lwaki Ebarasi, PhD [1], Jaakko Patrakka, MD, PhD [1]

SUPPLEMENTARY FIGURES

Supplementary Figure 1

Gene	RNA expression in Kidney (GTEx)	Signal in Glomeruli	Signal in Tubuli
Med1	2,7	Medium	Low
Med4	10,2	Medium	Low
Med6	4,2	Medium	Medium
Med7	3,9	n/a	n/a
Med8	13,3	negative	medium
Med9	8,8	negative	low
Med10	13,9	n/a	n/a
Med11	11,2	negative	negative
Med12	6,6	High	High
Med12L	0	negative	negative
Med13	3,9	n/a	n/a
Med13L	5,2	low	Medium
Med14	4,5	n/a	n/a
Med15	19,8	Medium	Low
Med16	14,4	low	high
Med17	2,8	n/a	n/a
Med18	3,9	negative	Low
Med19	6,9	low	Medium
Med20	4,5	Medium	negative
Med21	7,2	High	low
Med22	5,9	high	low
Med23	4,1	n/a	n/a
Med24	n/a	n/a	n/a
Med25	17,2	n/a	n/a
Med26	1,8	negative	negative
Med27	6,3	Medium	Medium
Med28	5,1	negative	negative
Med29	13,3	n/a	n/a
Med30	5,1	n/a	n/a
Med31	5,8	low	Medium
cdk8	1,9	n/a	n/a
cdk19	3,3	Medium	Medium
CCNC	23,5	Not detected	Medium

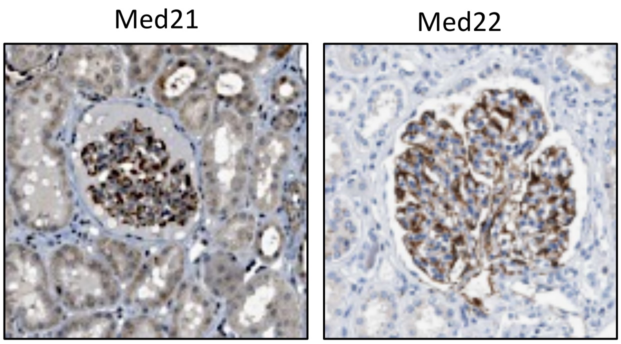

Source: www.proteinatlas.org

Supplementary Figure 2

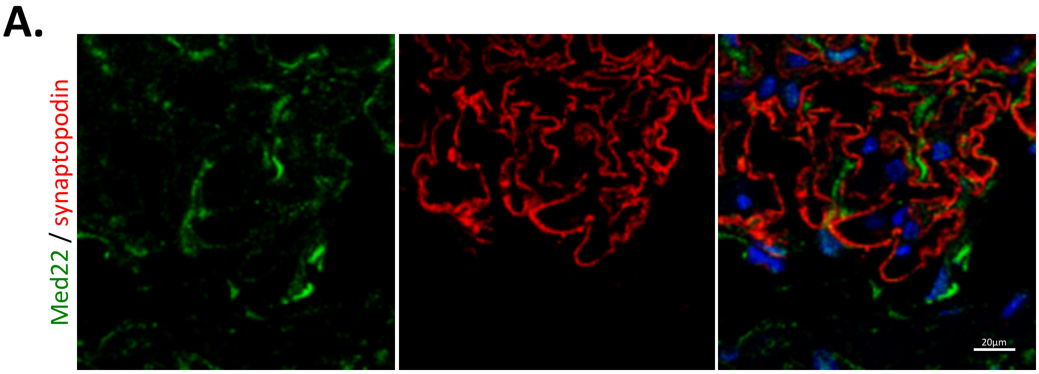

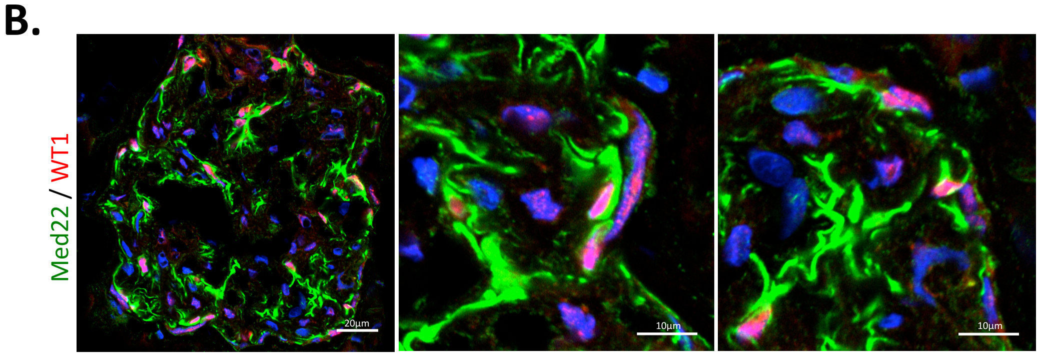

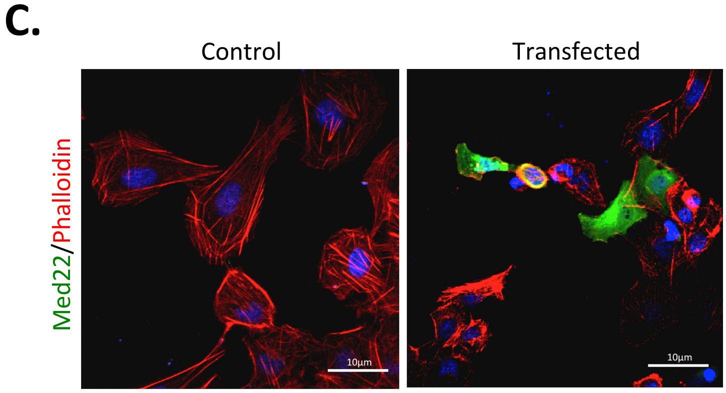

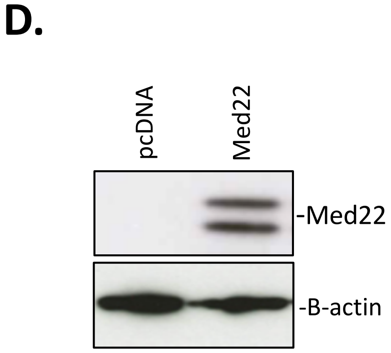

Supplementary Figure 3

A.

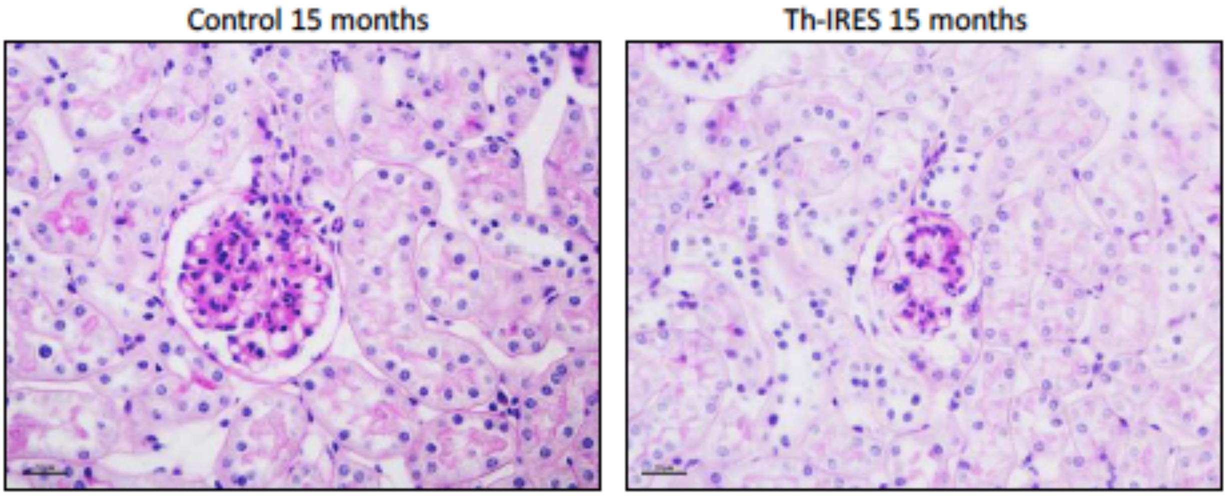

B

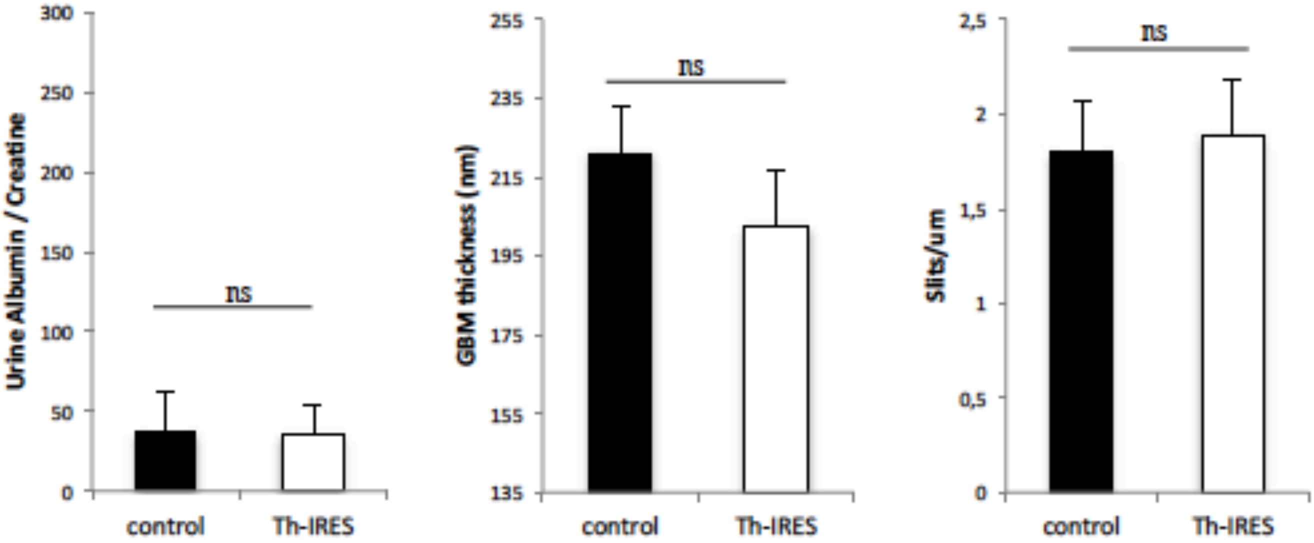

Supplementary Figure 4

A

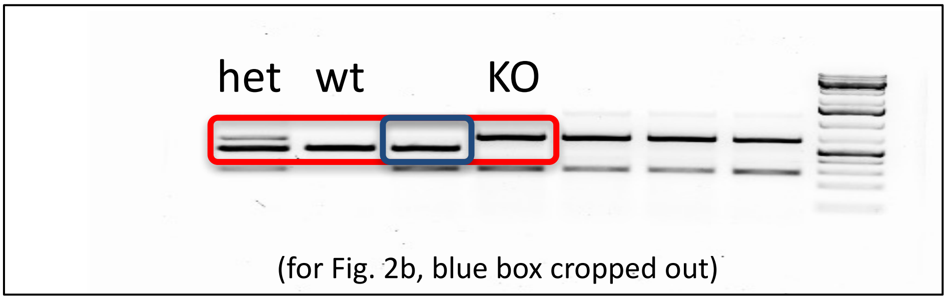

B

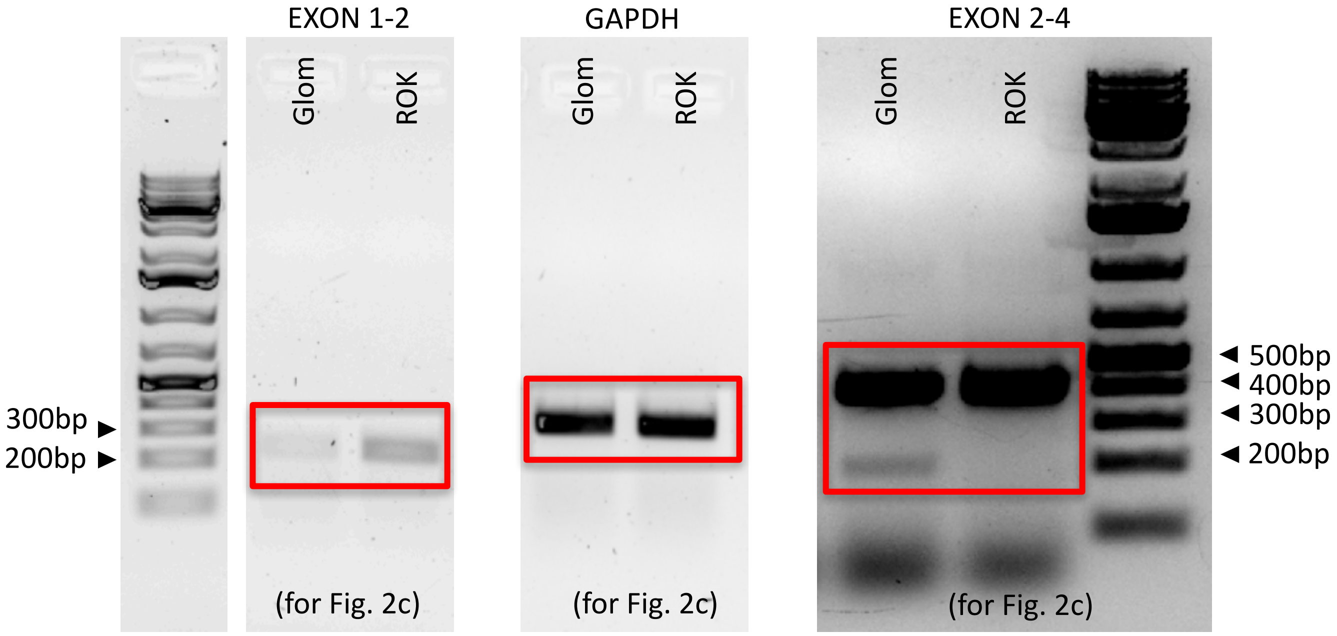

C

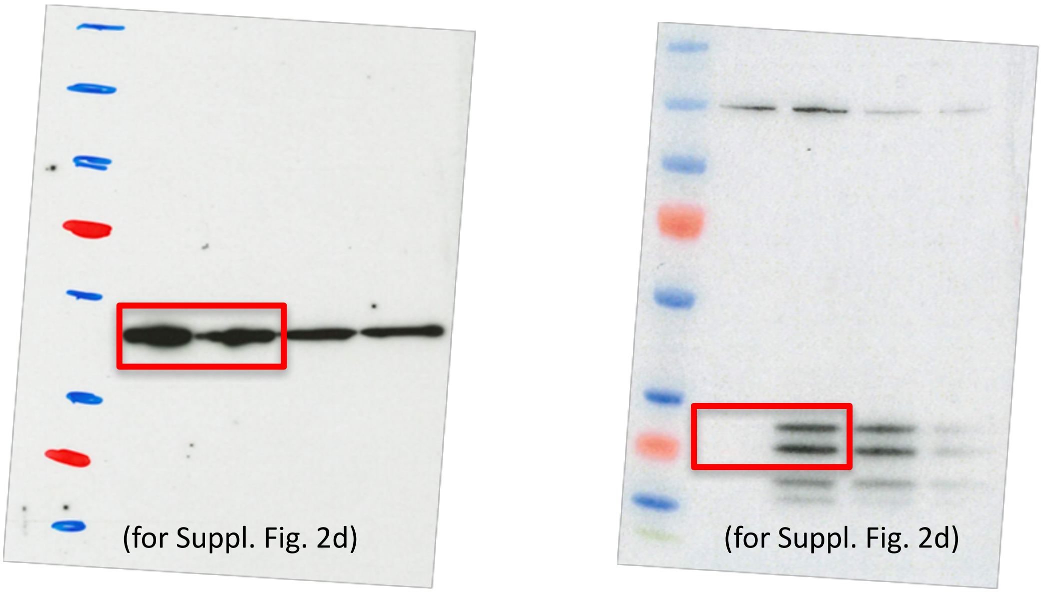

Supplement: Supplementary file 1 — Supplementary Figures. [file 41598_2020_76870_MOESM1_ESM.pdf]
