# Supplementary material for: Inactivation of mediator complex protein 22 in podocytes results in intracellular vacuole formation, podocyte loss and premature death
Source: Sci Rep. 2020 Nov 18;10:20037. doi: 10.1038/s41598-020-76870-0 (PMC7676236; doi:10.1038/s41598-020-76870-0)
Supplement: Supplementary file 2 — Supplementary Table 1. [file 41598_2020_76870_MOESM2_ESM.pdf]

SUPPLEMENTAL TABLE 1. RNAseq analysis of pod-Med22 glomeruli.

	CJP2133	I KJP2130	Control_mean	KO_mean	baseMean	log2FoldChange	stat	pvalue	padj
**Mgat5b**	1144,52	292,33	1629,16	314,23	1136,06	-1,18	-7,033	2,02E-12	5,25E-09
**Vtcn1**	510,50	160,64	478,63	138,14	350,95	-1,11	-6,897	5,30E-12	1,02E-08
**2610204G07Rik**	106,03	17,75	67,51	10,93	46,29	-0,91	-5,522	3,35E-08	2,87E-05
**Krt7**	3646,72	1497,14	3219,11	1593,89	2609,65	-0,91	-8,883	6,50E-19	5,01E-15
**Pvrl4**	696,33	230,69	611,79	225,73	467,01	-0,90	-5,598	2,17E-08	1,97E-05
**RP24-289J12.1**	3326,43	1798,81	3561,96	1876,61	2929,95	-0,88	-12,45	1,32E-35	2,03E-31
**Fgfbp1**	3036,74	1893,14	3510,24	1711,21	2835,60	-0,85	-6,634	3,27E-11	5,60E-08
**Slc26a7**	1465,90	963,85	1599,94	674,51	1252,90	-0,83	-5,274	1,33E-07	9,33E-05
**Gm15866**	81,99	20,55	58,09	19,14	43,48	-0,81	-4,848	1,25E-06	0,000550658
**Sptbn5**	2623,54	2174,26	4767,32	1761,89	3640,28	-0,76	-4,528	5,96E-06	0,001956212
**Gm4804**	87,45	45,76	91,72	28,60	68,05	-0,75	-4,457	8,31E-06	0,002481548
**PLA2G4B**	153,04	75,65	160,84	59,53	122,85	-0,74	-4,432	9,33E-06	0,002664392
**Xlr3b**	289,68	53,24	182,35	69,85	140,16	-0,74	-4,388	1,14E-05	0,002986058
**Opcml**	167,25	37,36	205,49	31,34	140,19	-0,73	-4,596	4,31E-06	0,001511158
**Gm826**	94,01	45,76	86,12	35,41	67,11	-0,71	-4,292	1,77E-05	0,004410347
**BC029214**	4277,46	2909,29	4751,94	2662,46	3968,39	-0,71	-5,982	2,20E-09	2,61E-06
**Axdnd1**	321,38	144,76	288,33	147,81	235,64	-0,70	-4,735	2,19E-06	0,000865971
**Myl7**	572,81	255,91	651,17	284,05	513,50	-0,67	-4,011	6,05E-05	0,012146546
**H2-Q6**	73981,53	35383,16	66252,81	29334,38	52408,40	-0,67	-4,021	5,80E-05	0,011921347
**Arhgap24**	19929,04	12485,21	18397,98	11192,63	15695,97	-0,66	-6,971	3,14E-12	6,93E-09

**2810433D01Rik**	538,92	391,33	684,38	377,64	569,36	-0,65	-4,599	4,25E-06	0,001511158
**Top1mt**	4767,18	3291,28	5680,10	3386,30	4819,93	-0,65	-5,95	2,69E-09	2,96E-06
**Gja1**	336,69	173,72	342,44	183,42	282,81	-0,64	-4,225	2,39E-05	0,005594011
**Med22**	2184,09	1500,88	2138,60	1350,84	1843,19	-0,61	-6,427	1,30E-10	2,00E-07
**1700029J07Rik**	185,83	116,75	186,28	99,39	153,70	-0,59	-3,68	0,00023	0,032707235
**Magi2**	10124,66	6750,68	10173,25	6530,96	8807,39	-0,59	-6,407	1,48E-10	2,08E-07
**6430573F11Rik**	188,02	161,58	218,09	112,99	178,68	-0,58	-3,548	0,00039	0,047473589
**Rab3b**	3988,87	2597,35	4811,46	2819,00	4064,29	-0,57	-3,913	9,12E-05	0,016632146
**Dkk2**	11872,59	11979,00	17300,96	9843,60	14504,45	-0,57	-3,729	0,00019	0,029129745
**Clic3**	8389,85	6082,90	9732,81	5799,91	8257,97	-0,56	-3,864	0,00011	0,018868801
**Kcnf1**	2484,71	1484,07	2096,99	1279,93	1790,60	-0,55	-3,913	9,10E-05	0,016632146
**Igfbp3**	1307,40	774,25	1182,43	747,21	1019,22	-0,53	-3,944	8,00E-05	0,01505039
**Nphs2**	117077,48	86610,06	114369,29	76240,43	100070,97	-0,53	-5,311	1,09E-07	8,12E-05
**Timp3**	53689,57	34662,14	47356,54	31875,22	41551,05	-0,51	-4,887	1,02E-06	0,000478024
**Trib2**	9167,07	7217,66	9317,39	6329,32	8196,86	-0,51	-5,308	1,11E-07	8,12E-05
**Tdrd5**	7733,97	5649,54	8136,89	5425,55	7120,14	-0,51	-4,395	1,11E-05	0,002986058
**Asb15**	2087,90	1261,78	1873,87	1228,02	1631,67	-0,50	-3,912	9,17E-05	0,016632146
**Ifngr1**	6279,00	4696,90	5910,26	4124,21	5240,50	-0,47	-4,53	5,89E-06	0,001956212
**Trabd2b**	4162,68	2116,36	4142,90	2792,57	3636,53	-0,46	-3,549	0,00039	0,047473589
**Synpo**	102856,83	75427,73	108257,02	75736,77	96061,93	-0,44	-3,676	0,00024	0,032813171
**Metrnl**	3649,99	2400,28	3706,78	2652,40	3311,39	-0,44	-4,456	8,37E-06	0,002481548
**Tmtc1**	6955,65	5442,20	7261,91	5336,89	6540,03	-0,41	-4,47	7,82E-06	0,002412371
**S1pr4**	424,14	302,60	409,19	300,05	368,26	-0,40	-3,754	0,00017	0,027370026
**Upp1**	907,31	658,44	798,24	607,29	726,63	-0,36	-3,631	0,00028	0,037789176

**Myo1d**	25754,38	20196,94	24824,05	19260,39	22737,68	-0,35	-4,958	7,13E-07	0,000366783
**Podxl**	380359,17	288338,46	361271,58	292242,37	335385,62	-0,29	-3,591	0,00033	0,042044813
**Sqstm1**	15982,80	12955,93	15545,35	12756,27	14499,44	-0,28	-4,738	2,16E-06	0,000865971
**Itm2b**	82852,36	70618,76	85496,89	73001,01	80810,93	-0,22	-3,745	0,00018	0,028088274
**Emc1**	2054,01	2334,91	2019,83	2356,74	2146,17	0,22	3,889	0,0001	0,017816211
**Arf3**	8960,47	9727,22	8770,91	10356,82	9365,63	0,23	3,719	0,0002	0,029129745
**Os9**	6791,68	7425,00	6618,02	7850,62	7080,25	0,24	3,598	0,00032	0,041280117
**Rcn2**	2589,65	3196,95	2595,25	3078,88	2776,61	0,24	4,135	3,55E-05	0,007943893
**Pam**	7111,97	7935,88	6840,61	8128,79	7323,68	0,24	3,992	6,54E-05	0,012896038
**Wls**	10104,99	12173,27	10208,52	12233,20	10967,78	0,26	5,188	2,13E-07	0,000131383
**Qsox2**	1758,86	2012,69	1739,71	2111,78	1879,24	0,27	3,721	0,0002	0,029129745
**Snrpa1**	637,30	777,06	645,23	787,06	698,41	0,27	3,652	0,00026	0,035239729
**Itfg1**	3842,39	4564,27	3731,92	4543,34	4036,20	0,28	4,97	6,70E-07	0,000360066
**Fam168a**	3484,93	4443,79	3555,77	4355,81	3855,78	0,28	4,762	1,92E-06	0,000800701
**Tmem184c**	1238,53	1625,09	1287,43	1626,54	1414,59	0,32	4,027	5,65E-05	0,011845865
**Cxadr**	2101,02	2339,58	2096,90	2656,28	2306,67	0,32	3,72	0,0002	0,029129745
**Hexim1**	4572,60	4896,76	4382,09	5622,08	4847,09	0,33	3,605	0,00031	0,040866891
**Sap18**	3561,45	3893,69	3120,31	3997,20	3449,14	0,33	3,719	0,0002	0,029129745
**Pde3b**	906,21	1126,36	944,44	1217,69	1046,91	0,34	3,565	0,00036	0,045541808
**Tmem242**	808,92	1083,40	821,92	1056,51	909,89	0,34	3,973	7,09E-05	0,013491218
**Ptprd**	4722,36	6107,18	5054,54	6657,17	5655,52	0,36	3,713	0,0002	0,029496292
**Nt5dc3**	650,42	769,58	609,22	801,16	681,20	0,36	3,865	0,00011	0,018868801
**R3hdm4**	2222,35	2568,40	2186,72	2886,94	2449,31	0,36	3,77	0,00016	0,025986785
**Cd99l2**	2716,45	3040,05	2565,49	3483,90	2909,89	0,40	3,99	6,61E-05	0,012896038

**Mgat4a**	4421,75	5757,88	4371,41	5958,83	4966,69	0,40	4,01	6,06E-05	0,012146546
**Gm16515**	2519,69	3420,17	2839,07	3883,36	3230,68	0,40	3,862	0,00011	0,018886446
**Axl**	14067,62	15912,85	12994,11	17513,93	14689,04	0,41	5,136	2,81E-07	0,000166622
**Sgcb**	681,03	921,82	658,90	909,47	752,86	0,41	3,629	0,00028	0,037789176
**Pcsk5**	2896,82	3940,39	2762,60	3773,61	3141,73	0,42	4,968	6,77E-07	0,000360066
**Pigs**	1276,79	1654,98	1346,12	1885,01	1548,21	0,42	3,72	0,0002	0,029129745
**Cd82**	5002,21	5628,06	4715,86	6614,53	5427,86	0,44	4,315	1,60E-05	0,004106385
**Sema3e**	6750,14	8988,45	6271,65	8853,82	7239,97	0,45	4,482	7,40E-06	0,002327729
**Gpr182**	1524,93	2349,85	1597,97	2327,27	1871,46	0,45	3,663	0,00025	0,03399878
**Crim1**	13036,79	15387,03	11790,49	16943,65	13722,92	0,46	4,059	4,93E-05	0,010563279
**Zc2hc1c**	154,13	208,27	144,55	209,76	169,00	0,46	3,817	0,00014	0,022155743
**Gm10535**	5,47	15,88	4,53	28,24	13,42	0,46	3,585	0,00034	0,042649381
**Col4a5**	10406,69	12650,52	9663,70	13642,31	11155,68	0,47	5,697	1,22E-08	1,25E-05
**Fgfr1**	5961,99	7433,41	6333,77	9538,79	7535,65	0,48	3,602	0,00032	0,040866891
**Olfml2a**	241,58	372,65	273,79	432,56	333,33	0,52	3,692	0,00022	0,031778922
**Npnt**	33626,08	41438,04	30847,69	45640,60	36395,03	0,52	5,443	5,24E-08	4,25E-05
**Ust**	636,21	622,02	528,85	857,07	651,93	0,52	3,554	0,00038	0,047271012
**Morn4**	520,33	792,00	490,44	768,31	594,64	0,52	3,902	9,53E-05	0,017083325
**Lsr**	115,87	174,65	125,50	204,16	155,00	0,53	3,675	0,00024	0,032813171
**Ptprv**	174,90	995,60	329,80	3395,05	1479,27	0,53	4,25	2,14E-05	0,005226875
**Ifi30**	560,78	697,67	555,15	887,99	679,97	0,53	3,848	0,00012	0,01971885
**Ap1s3**	127,90	174,65	104,92	172,32	130,20	0,54	3,723	0,0002	0,029129745
**D030028A08Rik**	118,06	194,26	130,12	208,02	159,33	0,57	4,619	3,85E-06	0,001413132
**Nxpe4**	28,42	74,72	32,54	144,71	74,60	0,57	3,879	0,0001	0,018160209

**Dok2**	165,06	254,97	158,65	269,67	200,28	0,59	4,233	2,30E-05	0,005464135
**Ptgfr**	402,28	600,54	349,75	757,97	502,83	0,61	3,603	0,00031	0,040866891
**AI429214**	110,41	159,71	103,21	185,98	134,25	0,61	3,984	6,76E-05	0,013039069
**Fbxl16**	92,92	165,31	109,79	210,05	147,38	0,61	3,786	0,00015	0,024552568
**Csdc2**	777,22	1260,85	898,01	1667,25	1186,47	0,61	3,88	0,0001	0,018160209
**Slc37a2**	396,81	908,75	538,77	1138,99	763,85	0,62	3,688	0,00023	0,031984936
**Col26a1**	189,11	279,25	192,98	355,50	253,92	0,62	4,026	5,68E-05	0,011845865
**Tmem54**	1452,78	2212,56	1456,45	2512,61	1852,51	0,62	4,52	6,17E-06	0,001983482
**Chrd**	61,22	124,22	75,51	135,66	98,07	0,62	4,22	2,44E-05	0,00561414
**Ssc5d**	197,86	298,87	178,05	316,42	229,94	0,63	4,39	1,13E-05	0,002986058
**Tspan2**	4863,38	8911,87	4846,48	8203,53	6105,37	0,63	5,069	3,99E-07	0,000227946
**Arhgap20**	18,58	58,84	37,08	152,96	80,53	0,64	4,113	3,90E-05	0,008597095
**Scg5**	114,78	234,42	152,80	291,06	204,65	0,64	4,072	4,67E-05	0,010136608
**Wnt8b**	54,66	113,01	65,37	162,61	101,84	0,64	3,804	0,00014	0,023113838
**Rapgefl1**	69,96	101,80	71,74	137,20	96,29	0,66	4,239	2,25E-05	0,005419073
**Anks1b**	455,84	707,94	427,94	758,55	551,92	0,66	4,917	8,80E-07	0,000424126
**Masp1**	4,37	18,68	8,13	32,10	17,12	0,67	4,195	2,72E-05	0,006178088
**Ighv9-3**	10,93	45,76	6,84	34,64	17,26	0,67	4,397	1,10E-05	0,002986058
**6030408B16Rik**	28,42	41,09	34,70	134,16	72,00	0,69	4,301	1,70E-05	0,004291909
**Abat**	3233,51	5591,63	3402,18	6121,88	4422,06	0,69	5,225	1,74E-07	0,000112133
**Col4a6**	541,10	734,09	557,57	1113,28	765,96	0,69	4,428	9,50E-06	0,002665153
**Asic2**	406,65	680,86	479,42	954,43	657,55	0,70	4,533	5,83E-06	0,001956212
**Aplp1**	6074,58	10353,91	6042,91	12152,61	8334,05	0,71	4,651	3,30E-06	0,001244254
**Polr2a**	9824,05	13399,56	9396,92	17066,53	12273,02	0,73	6,035	1,59E-09	2,04E-06

**Il12rb1**	4,37	45,76	9,26	41,31	21,28	0,74	4,651	3,31E-06	0,001244254
**Frem1**	79,80	151,30	102,43	307,08	179,17	0,75	4,45	8,58E-06	0,002496342
**Cyp1b1**	818,76	1365,45	722,26	1377,04	967,80	0,75	5,63	1,81E-08	1,74E-05
**Tmod2**	80,89	121,42	66,59	147,76	97,03	0,77	4,866	1,14E-06	0,000515706
**Zdhhc2**	142,11	359,58	191,90	474,53	297,89	0,81	4,936	7,98E-07	0,000396847
**Kirrel2**	297,33	546,37	344,52	1046,36	607,71	0,81	4,81	1,51E-06	0,000647824
**Tshb**	47,01	99,00	34,97	98,31	58,72	0,87	5,264	1,41E-07	9,45E-05
**Dusp14**	88,54	216,68	93,76	264,94	157,95	1,07	7,031	2,04E-12	5,25E-09
**Fndc5**	526,89	1174,92	464,58	1484,78	847,16	1,23	8,251	1,57E-16	6,05E-13
**Efemp1**	703,98	1501,81	677,09	2195,43	1246,47	1,25	8,432	3,40E-17	1,75E-13
