# Supplementary material for: Inactivation of mediator complex protein 22 in podocytes results in intracellular vacuole formation, podocyte loss and premature death
Source: Sci Rep. 2020 Nov 18;10:20037. doi: 10.1038/s41598-020-76870-0 (PMC7676236; doi:10.1038/s41598-020-76870-0)
Supplement: Supplementary file 5 — Supplementary Legends. [file 41598_2020_76870_MOESM5_ESM.docx]

**SUPPLEMENTARY FIGURES**

**Supplemental Figure 1. Summary of mediator subunit expression in Human Protein Atlas (www.proteinatlas.org).** Whereas all mediator subunits show expression in kidney tissue by RNA sequencing, only Med21 and Med22 show high staining intensity in glomeruli and low signal in tubuli. Magnifications x60

**Supplemental Figure 2. Expression of Med22 in the glomerulus and transfected cells.** (A) Med22 (green) does not co-localize with foot process marker synaptopodin (red) in human glomeruli as detected by double immunofluorescence staining. **(**B) Med22 (green) does not co-localize with WT1 (red) in human glomeruli as detected by double immunofluorescence staining, suggesting localization to the cytoplasm of podocytes. (C) In differentiated podocytes (control) no Med22 staining is detected (left). In transfected cultured podocyte cells, Med22 (green) is detected in the cytoplasm. The cells were co-stained with DAPI and phalloidin. (D) In Western blotting of transfected human podocyte cells, two bands around 22kD are detected, whereas control (pcDNA) cells do not show any reactivity. $\beta$-actin was used as a loading control. Magnifications x60 and x200.

**Supplemental Figure 3. Haploinsufficiency for Med22 does not result in any obvious renal phenotype. (**A) Histology of 15month old Med22 TH-IRES heterozygous mice show no renal histological changes in comparison to littermate controls. (B) Albuminuria as measured by urine albumin/creatinine ratios, GBM thickness, and foot process width as measured by slits/GBM length are similar in 15-month-old Th-IRES heterozygotes and control mice.

**Supplemental Figure 4. Original images.** (A) PCR-based genotyping of pod-Med22 mice (cropped image in Fig. 2B). (B) A single cDNA fragment spanning from exon 2 to exon 4 Med22 mRNA is amplified in wild type animals. In pod-Med22 mice, we detect a shorter variant in the glomerulus corresponding in size to skipping of exon 3 (cropped image in. Fig 2C). **(**C) Western blotting of cultured podocytes for Med22 (cropped image in Suppl. Fig 2D).

**Supplemental media 1 and 2: Analysis of podocyte vacuoles in expanded kidney samples.** Podocytes exhibit vacuoles that are surrounded by cytoplasmic tdTomato in pod-Med22 mice. No obvious connections to extracellular space are observed.
